# Enabling Genomics Pipelines in Commodity Personal Computers With Flash Storage

**DOI:** 10.3389/fgene.2021.615958

**Published:** 2021-04-29

**Authors:** Nicola Cadenelli, Sang-Woo Jun, Jordà Polo, Andrew Wright, David Carrera, Arvind

**Affiliations:** ^1^Barcelona Supercomputing Center (BSC), Barcelona, Spain; ^2^Computer Science Department, University of California, Irvine, Irvine, CA, United States; ^3^Computer Science and Artificial Intelligence Laboratory, Massachusetts Institute of Technology (MIT), Cambridge, MA, United States

**Keywords:** precision medicine, N-grams, K-mers, flash storage, NVME, asynchronous key-value store

## Abstract

Analysis of a patient's genomics data is the first step toward precision medicine. Such analyses are performed on expensive enterprise-class server machines because input data sets are large, and the intermediate data structures are even larger (TB-size) and require random accesses. We present a general method to perform a specific genomics problem, *mutation detection*, on a cheap commodity personal computer (PC) with a small amount of DRAM. We construct and access large histograms of k-mers efficiently on external storage (SSDs) and apply our technique to a state-of-the-art reference-free genomics algorithm, SMUFIN, to create SMUFIN-F. We show that on two PCs, SMUFIN-F can achieve the same throughput at only one third (36%) the hardware cost and half (45%) the energy compared to SMUFIN on an enterprise-class server. To the best of our knowledge, SMUFIN-F is the first reference-free system that can detect somatic mutations on commodity PCs for whole human genomes. We believe our technique should apply to other k-mer or n-gram-based algorithms.

## 1. Introduction

As genome sequencing becomes cheaper and more available, analysis of *somatic mutations* has become an essential tool in the study and treatment of cancer. Somatic mutations are mutations acquired by an organism instead of inherited from a parent, and they are identified by comparing genomes of healthy cells and tumoral cells of the same organism. This results in more accurate identification of mutations involved in oncogenesis, or the development of tumors. The knowledge of the mutations present in cancerous tumors can be used to predict the types of cancer a patient may develop, leading to effective and personalized treatments.

A leading algorithm and implementation for detecting somatic mutations is SMUFIN (Moncunill et al., 2014; Cadenelli et al., 2017), which detects both point and structural somatic mutations without full genome reconstruction or alignment against a reference. Since SMUFIN is *reference-free*, it avoids costly alignment which often takes up to 70% of genome analysis pipeline execution times (Wu et al., 2019). Additionally, by not aligning against a reference genome, SMUFIN can detect complex structural variations more effectively (Moncunill et al., 2014), work on species where reference genomes may be incomplete or unavailable (Leggett and MacLean, 2014), and even identify viruses.

One issue with SMUFIN is that, for a pair of typical human genome samples, it generates more than a terabyte of intermediate data structures, and takes over 12 h to complete on a reasonably powerful enterprise-class server with a 24-core Xeon CPU and 512 GB of DRAM. If we restrict the memory to 32 GB of DRAM, SMUFIN does not complete in a reasonable amount of time, i.e., 6–7 days! This performance degradation makes SMUFIN impractical for ordinary PCs.

We present a method in this paper to run the general genomics pipeline embedded in SMUFIN on PCs by exploiting external flash storage, which is a lot cheaper and cooler than DRAM. Of course the main challenge in using flash technology is that its access latency and granularity is several orders of magnitude higher than DRAM. New emerging storage technologies, e.g., 3DXPoint, have lower latency than flash SSDs but cost significantly more. The idea of multiple tiers of memory, from faster and expensive to slower and cheaper, is so intrinsic in computer systems that the concern of reducing the memory footprint to use cheaper secondary storage is likely to remain relevant in the foreseeable future.

This paper describes SMUFIN-F, a modification of the SMUFIN algorithm, that provides the same output as SMUFIN but in which all the big data structures reside in secondary storage (NAND-flash SSDs). SMUFIN-F is designed to overcome flash storage's four orders of magnitude longer latency and two orders of magnitude larger access granularity than DRAM. We deal with these overheads by restructuring both the creation of the intermediate data structures and the way they are referenced. We borrow ideas from GraFBoost (Jun et al., 2018), specifically the Sort-Reduce method, to make both inter-page and intra-page accesses to flash storage more sequential. Once the intermediate data are constructed, we use application-specific information to execute extremely efficient in-memory caching. Furthermore, SMUFIN-F is also optimized for newer NVMe devices by extensively using *asynchronous I/O* to hide access latency, and using 4KB random reads to minimize read amplification. The techniques we have used in SMUFIN-F can be used broadly in other k-mer or n-gram applications.

We show that when we reduce the DRAM from 512 to 32 GB, not only does SMUFIN-F complete execution within a reasonable amount of time, but it takes only 1.24x longer than the original SMUFIN using the full 512 GB. We also show that on an affordable commodity PC with a 6-core i7 CPU and 32 GB of memory, SMUFIN-F takes 1.87x longer than SMUFIN on the costly server. Alternatively, we can say that SMUFIN-F can achieve the same throughput (patients/hour) on two commodity PCs as SMUFIN on one enterprise server. Since one commodity PC costs 18% of the enterprise server and consumes only 45% of the energy, SMUFIN-F on two PCs provides the same throughput as SMUFIN at one third (36%) the capital cost and less than half (45%) the operational cost (energy). We believe that such improvements in the delivery cost will be essential to foster personalized medicine.

### 1.1. Related Work

Section 1.1.1 compares the SMUFIN method (not SMUFIN-F implementation) with other genomics pipelines and software. The section highlights how other methods tend to be specialized to identify specific variations and how other reference-free methods also require significant computational and memory resources; so much so that some of these methods only target one human chromosome. Furthermore, this section compares the k-mer counting algorithm used in SMUFIN to other k-mer implementations that also aim to reduce their main memory footprint. Section 1.1.2 analyzes the similarities and differences of some of the ideas and optimizations used in SMUFIN-F and in other flash-based databases.

#### 1.1.1. Genomics and K-mer Counting

Methods to find mutations typically align reads from a sequenced sample to a reference genome. Some of these methods (Lam et al., 2008; Li and Durbin, 2009), usually run on powerful servers for faster execution but, generally, do not use a lot of memory. However, somatic mutations, mutations that occur after birth, are particularly challenging because they usually involve comparing normal and tumoral samples from the same patient, and reads carrying variations are harder to align (Degner et al., 2009). Current reference-based approaches tend to be very specialized and use different algorithms to target a particular kind of variant (de Ridder et al., 2014). For instance, some (Cibulskis et al., 2013; Rimmer et al., 2014; Peterlongo et al., 2017; Prezza et al., 2020) are designed for single nucleotide variants (SNV) and INDELs. Others (Chen et al., 2009; Ye et al., 2009; Stütz et al., 2012), instead, are designed for structural variants (SV) with different characteristics. Hence, defining a complete catalog of variations generally requires complex pipelines with combinations of multiple methods.

Emerging reference-free methods have the potential to provide more accurate results, but they also require significant computational and memory resources. Methods to detect single nucleotide polymorphisms (SNP) based on De Bruijn graphs (Salikhov et al., 2013) easily exceed the memory of a server with 512 GB of DRAM (Iqbal et al., 2012; Leggett et al., 2013; Nordström et al., 2013). For instance, the processing of a single human chromosome (out of 23) can require as much as 105 GB (Uricaru et al., 2015); implying that whole human genome processing would require much higher amounts of memory. Some methods use a cascade of Bloom filters to represent De Bruijn graphs (Compeau et al., 2011; Chikhi and Rizk, 2013) and manage to keep a significantly lower memory footprint (Uricaru et al., 2015). As these variant calling methods target a particular kind of non-somatic mutation they are limited in scope. SMUFIN is different in that since it is a comprehensive reference-free method that targets somatic mutations, all kinds of variants, from SNVs to large SVs. Furthermore, SMUFIN can also be used to identify viruses.

Counting the frequencies of k-mers is an algorithm that is widely used in many areas of genomics (Xiao et al., 2018); from genome assembly and error detection to sequence alignment and variant calling (Kelley et al., 2010; Li et al., 2010). Others (Marçais and Kingsford, 2011; Rizk et al., 2013; Audano and Vannberg, 2014; Deorowicz et al., 2015; Li and Yan, 2015; Jiang et al., 2019) have explored ways to optimize k-mer counting with reduced memory and storage. While these k-mer counting algorithms process a single sample, SMUFIN processes k-mer counters of normal and tumoral samples of the same patient together, potentially making the memory footprint even bigger. Finally, this work goes beyond the mere k-mer counting algorithm, and it considers a complete genomics application that needs to access the k-mers histogram. For this reason, the work presented in this paper can also be applied to other genomics applications that rely on k-mers; including assembly-based variant calling and graph-based *de-novo* assembly.

#### 1.1.2. Flash Storage for Databases

As section 3.3 details, one of the core ideas of SMUFIN-F is to reduce the memory footprint using flash memory. Flash memory can improve database and key-value (KV) store performance transparently by providing faster I/O compared to mechanical disks (Lee et al., 2008, 2009; Bausch et al., 2011). However, its true potential is achieved using algorithms (Jung et al., 2012; Lee et al., 2012; Chen and Ordonez, 2013; Kanza and Yaari, 2016) and data structures (Agrawal et al., 2009; Shi et al., 2013; Jin et al., 2016; Sadoghi et al., 2016) that are aware of underlying flash characteristics. Thanks to the high bandwidth and low latency of flash storage compared to magnetic disks, databases benefit from using them as a cache layer between memory and disk (Do et al., 2011; Kang et al., 2012, 2016). Many modern production databases have been designed to take advantage of the high bandwidth provided by flash storage (Weil et al., 2006; Nath and Kansal, 2007; Lim et al., 2011; ScyllaDB, 2017, 2019; Kourtis et al., 2019). One of the most prominent databases optimized for fast storage such as flash is RocksDB (2019), which is a widely used open-source key-value store. MyNVM (Eisenman et al., 2018) extends MyRocks (2019)—a MySQL storage engine that integrates with RocksDB—to use a second-layer NVM cache.

Others, like ScyllaDB (2019, 2017) and uDepot (Kourtis et al., 2019), are key-value stores built from the bottom-up to deliver the performance of NVM, using a task-based design to support asynchronous I/O.

Other key-value stores focus on reducing the memory footprint of the indexes by doing multiple storage accesses to storage, but that generally increases the look-up latency. FAWN (Andersen et al., 2009) is a distributed KV store that uses an in-memory hash index to store only a fragment of the actual key. This reduces the memory requirement but introduces the chance of requiring two reads from flash. Similarly, FlashStore (Debnath et al., 2010) stores compact key signatures instead of full keys to trade RAM usage with false positive flash read operations. BloomStore (Lu et al., 2012) uses an index structure based on Bloom filters to efficiently store all indexes in flash storage. SkimpyStash (Debnath et al., 2011) uses a hash table directory in DRAM to index key-value pairs stored in a log-structure on flash, and to use less than one pointer per key it moves most of the pointers from DRAM to flash using chains of key-value entries. Here a look-up might translate to multiple flash look-ups to traverse the chain. Differently from how section 3.3 shows, our key-value store leverages the fact that the entries are sorted to reduce the size of the index. Besides, since the look-up time is critical, our key-value store does not admit multiple flash reads per look-up by design, but due to the usage of Bloom filters, it admits false positives.

The rest of the paper is organized as follows: section 2 describe the hardware equipment, the methodology, and the reference genome used to compare SMUFIN-F against SMUFIN. Section 3 describes the method, presenting the proposed SMUFIN-F. Section 4 evaluates the results obtained with SMUFIN-F. Finally, section 5 concludes with a discussion of the work presented.

## 2. Equipment

This section describes the hardware configuration, the methodology, and the reference used to evaluate SMUFIN-F against the state-of-the-art. Besides, the section offers a performance comparison of the baseline version of SMUFIN on various costly enterprise-class server machines with 100s of GB of DRAM against SMUFIN-F on a cheaper commodity PC with flash storage. Table 1 summarizes the different hardware configurations.

**Table 1:** Experimental setups.

	**Marenostrum 4**	**FatNode**	**Commodity & CommodityNVMe**
Type	HPC/Enterprise server	Enterprise server	Commodity PC
CPU	2x Xeon Platinum 8160 @2.1 GHz (approx. 9,400 USD)	2x Xeon E5-2680 v3 @2.50 GHz (approx. 3,500 USD)	Core i7-8700K @3.70 GHz (approx. 350 USD)
# CPU threads	2x 24	2x 24	12
DRAM	384 GB, 12x 32 GB DDR4-2667 (approx. 4,200 USD)	512 GB, 16x 32 GB DDR4-2133 (approx. 3,300 USD)	32 GB, 4x 8 GB DDR4-2133 (approx. 200 USD)
Storage	[-25mm] 14 PB of GPFS elastic storage system (unknown cost)	[-20mm] 4x 1.5 TB Intel DC P3608 PCIe NVMe SSDs 4x 850 K random 4 KiB IOPS (approx. 2,400 USD)	**Commodity** 4x 1 TB SATA-III Samsung 860 EVO 4x 98 K random 4 KiB IOPS (approx. 600 USD) **CommodityNVMe** 2x 1TB NVMe Samsung 970 EVO Plus 2x 620 K random 4 KiB IOPS (approx. 500 USD)
Estimated cost	13,600 USD (without storage)	9,200 USD	1,150 USD or 1,650 USD

### 2.1. Evaluation Setup and Methodology

In “Marenostrum 4,” we execute only the baseline version of SMUFIN. This system is a multi-node HPC (high performance computing) system and the current production environment where the application runs with multiple partitions; each in a different node. In “FatNode,” an enterprise 2U server, we execute the baseline, using all the 512 GB of DRAM, and SMUFIN-F, capping its DRAM budget with cgroups to only 31 GB (leaving 1 GB out of our 32 GB budget for OS and other background software) and using four PCIe NVMe storage devices. We use this system to show the impact of reducing the DRAM budget, while using the same CPU. In “Commodity,” a normal commodity PC with only 32 GB of DRAM and four SATA-III SSDs, we execute SMUFIN-F to demonstrate how it can run the full SMUFIN software pipeline on much cheaper PCs. We also evaluate “CommodityNVMe,” which augments Commodity with two M.2 PCIe NVMe storage devices to accelerate random accesses in the Label unit. Commodity and CommodityNVMe represent a potential commodity PC that one can find in a lab or in a medical practitioner's desk. In both FatNode and Commodity, storage is organized into a software RAID-0 using a Linux md driver. While the power and energy consumption of Marenostrum 4 and FatNode were collected via IPMI (without accounting for air cooling and GPFS), the consumption of Commodity was measured using a power meter.

For each combination of system and implementation, we use different numbers of partitions in order to fit the working set in the available DRAM budged for each system. Unless differently specified, we report the aggregated time- and energy-to-solution metrics of all partitions executed sequentially.

### 2.2. Reference Genome

In each execution, we use the same parameters and we process the same personalized genome based on the Hg19 reference. This genome is characterized by randomly chosen germlines and somatic variants as described in Moncunill et al. (2014). The normal and tumoral samples are stored in gzip compressed FASTQ files that total 312 GB in size and grow to around 740 GB once uncompressed. Since improving the quality of results of the SMUFIN method is out of the scope of this work, the final output of SMUFIN and SMUFIN-F are exactly the same.

## 3. Method

Section 3.1 introduces the mutation detection problem, first abstractly, and then within the context of genomics. Section 3.2 describes the SMUFIN implementation and its limitations. Finally, section 3.3 presents the proposed method: SMUFIN-F; our modifications to SMUFIN that reduce the required system resources while offering the same and exact results.

### 3.1. Detecting Mutations: A Generalized Problem Formulation

N and T are two vary large sequences of characters, where N is a random sequence, and T is a mutated version of N produced by changing, inserting, and deleting potentially large sequences in a (small) number of places. Given the complete sequences N and T, we can easily perform a *diff* of the two sequences to detect the mutations used to produce T. Unfortunately, we are given only sampled versions of N and T, i.e., sets *R*_*N*_ and *R*_*T*_ each containing subsequences of N and T respectively. From *R*_*N*_ and *R*_*T*_, we want to detect the mutations used to produce T and show the local context of each mutation in N and T.

Let us assume the subsequences, or *reads*, in *R*_*N*_ and *R*_*T*_ are of uniform length of *r*, and collectively cover each location in N and T α times on average. If two reads in a read set (*R*_*N*_ or *R*_*T*_) have the same *k*-length character sequence, or *k-mer*, then the two reads either cover the same location in the original string, or the k-mer appears in multiple locations in the original string.

An important component of this fact is that *k* needs to be long enough so that the number of occurrences of a k-mer is small enough to be manageable.

A k-mer that exists only in *R*_*N*_ or only in *R*_*T*_, must correspond to a mutation site or a mutation itself. Because it appears only in one set, such a k-mer cannot be used to align normal and tumoral reads even though it covers the mutation site or the mutation itself. For the alignment of reads across *R*_*N*_ and *R*_*T*_, we look for *almost-matching* k-mers, that is, k-mers where the middle *k* − 2 characters match, but at least one of the end characters differs. As long as *k* is large enough, the middle *k* − 2 characters of the almost matching k-mer will only appear in a single location in N and T. With these almost-matching k-mers, which we call *interesting k-mers*, reads from N and T can be aligned together to determine the mutation site, a.k.a., the *break-point*, and the structure of the complete mutation. The detailed process of mutation detection using *interesting k-mers* will be introduced in section 3.2.

#### 3.1.1. Read Errors

If the process of producing *R*_*N*_ and *R*_*T*_ from N and T is noisy, then there is a chance of seeing *read errors*, i.e., characters in reads that do not match the corresponding characters in N and T. These read errors may cause some sequences to be incorrectly classified as mutations. Since read errors are unavoidable, the algorithm must deal with it systematically. For example, the presence of read errors requires more reads to cover each location for accuracy. Thus, with enough *coverage* α, if a k-mer is seen in only one read then it is likely the result of a read error. For us to have confidence that the detected mutation is not just a read error, interesting k-mers must be seen multiple times in *R*_*N*_ and/or *R*_*T*_. *k* also needs to be small enough so that the chance of read errors per k-mer is low enough to be useful.

#### 3.1.2. Applications to Genomics

With human DNA, the strings N and T are genomes which each consist of approximately 6 billion characters from the four character alphabet {A, C, G, T}. DNA is structured in a double helix containing complementary base pairs on the two strands with the pairings < A,T> and < C,G>. When a mutation is found in one strand of the helix, its complement is found in the other strand. Therefore, N and T can be treated as approximately 12 billion character sequences where half of the sequence is the complement of the other half.

N and T are sampled by taking DNA from normal cells and DNA from tumor cells, respectively. This process is not noise-free due to sequencing errors and possible samples from T that are contaminated with samples from N. The read sets *R*_*N*_ and *R*_*T*_ are produced by sequencing machines which read DNA by breaking apart the helix, cutting the strands into chunks, and producing reads on the order of tens to a few hundreds of characters for next generation sequencing. The reads are always performed in a deterministic direction in the two strands of the DNA, but the two strands are read in opposite directions, so a read of ATCCG on one strand corresponds to its reverse complement, CGGAT, on the other strand. These reverse complement k-mers can be aligned with each other during mutation reconstruction to effectively double the read coverage of the genome.

Due to the noise in *R*_*T*_, the process of selecting interesting k-mers becomes more like a heuristic process than a set of objective criteria. A k-mer that appears only once in *R*_*N*_ or *R*_*T*_ is more likely to be a read error than a mutation. Therefore, the number of times that k-mer is seen in *R*_*N*_ or *R*_*T*_ must be considered when determining if it is interesting. The heuristic process for selecting interesting k-mers can vary greatly depending on the intended use. For example, in a research setting it is beneficial to liberally mark k-mers as interesting to get as much information about potential mutations as possible.

While a lot of human DNA appears nearly random, there are some long sequences that appear in many places within the DNA. If a k-mer appears in multiple locations and it has the same local context, i.e., it is part of a larger repetitive sequence, then there is no change necessary to the algorithm. Fortunately this is very rare for our chosen *k* and can be ignored.

### 3.2. Existing SMUFIN Implementations

SMUFIN (Moncunill et al., 2014; Cadenelli et al., 2017) is an algorithm for mutation detection between healthy and tumor genomes. The end goal of SMUFIN is to reveal the exact mutations found in a tumorous sample, therefore allowing doctors to produce personalized medicine for those specific mutations. In its current state, SMUFIN is a research tool used by scientists, and as a result, it is desirable for SMUFIN to produce and keep more data to create a more complete picture of the potential mutations seen in the genome. The analysis in this paper assumes settings that biologists have used for exploratory research so far. In the future, different settings may be used for clinicians using SMUFIN as a tool to produce personalized medicine.

**Figure 1:**
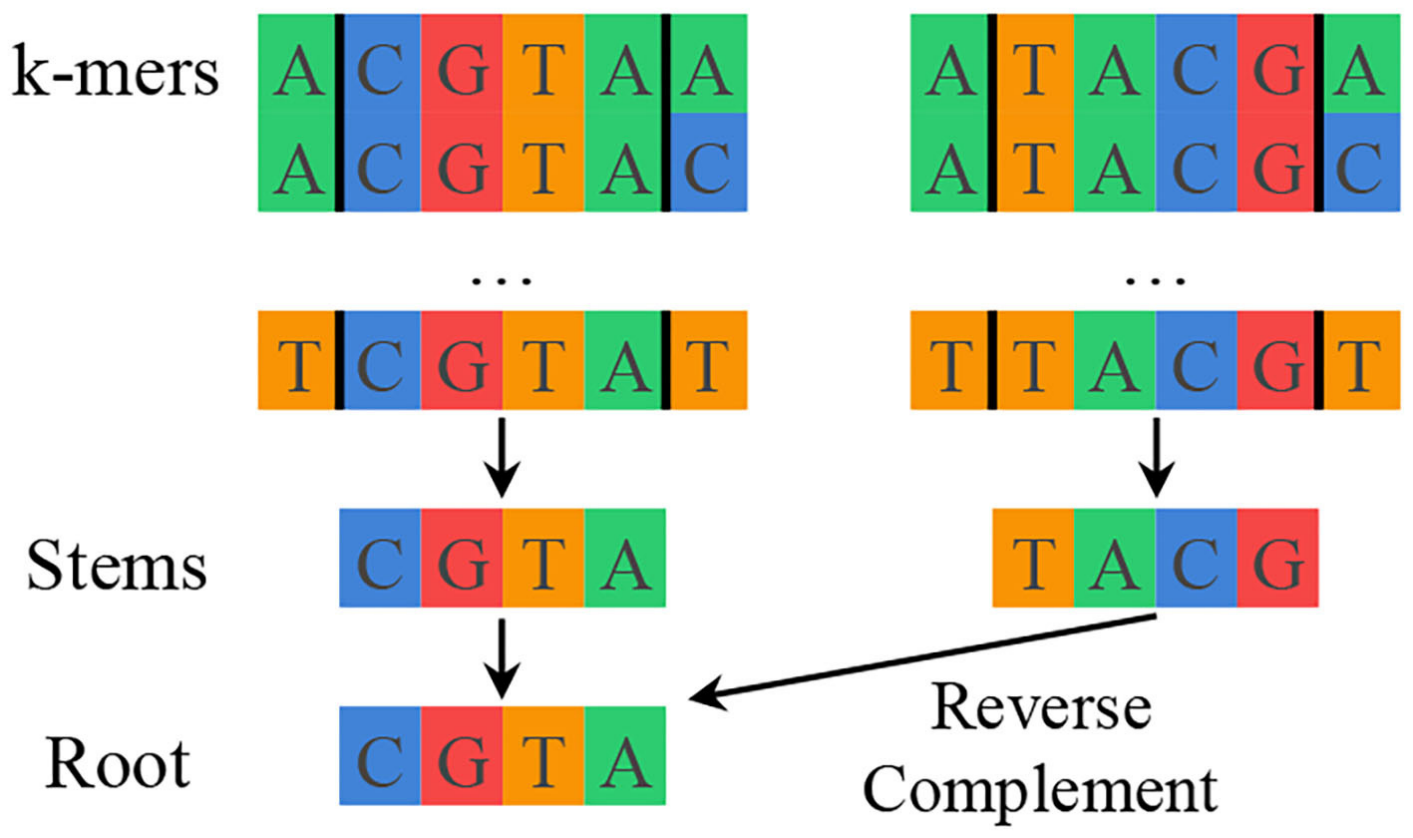
Hierarchy of k-mers, stems, and roots showing all the k-mers that map to the root CGTA. There are (4 · 4=) 16 different k-mers that belong to a stem, and two different stems that belong to a root.

**Figure 2:**
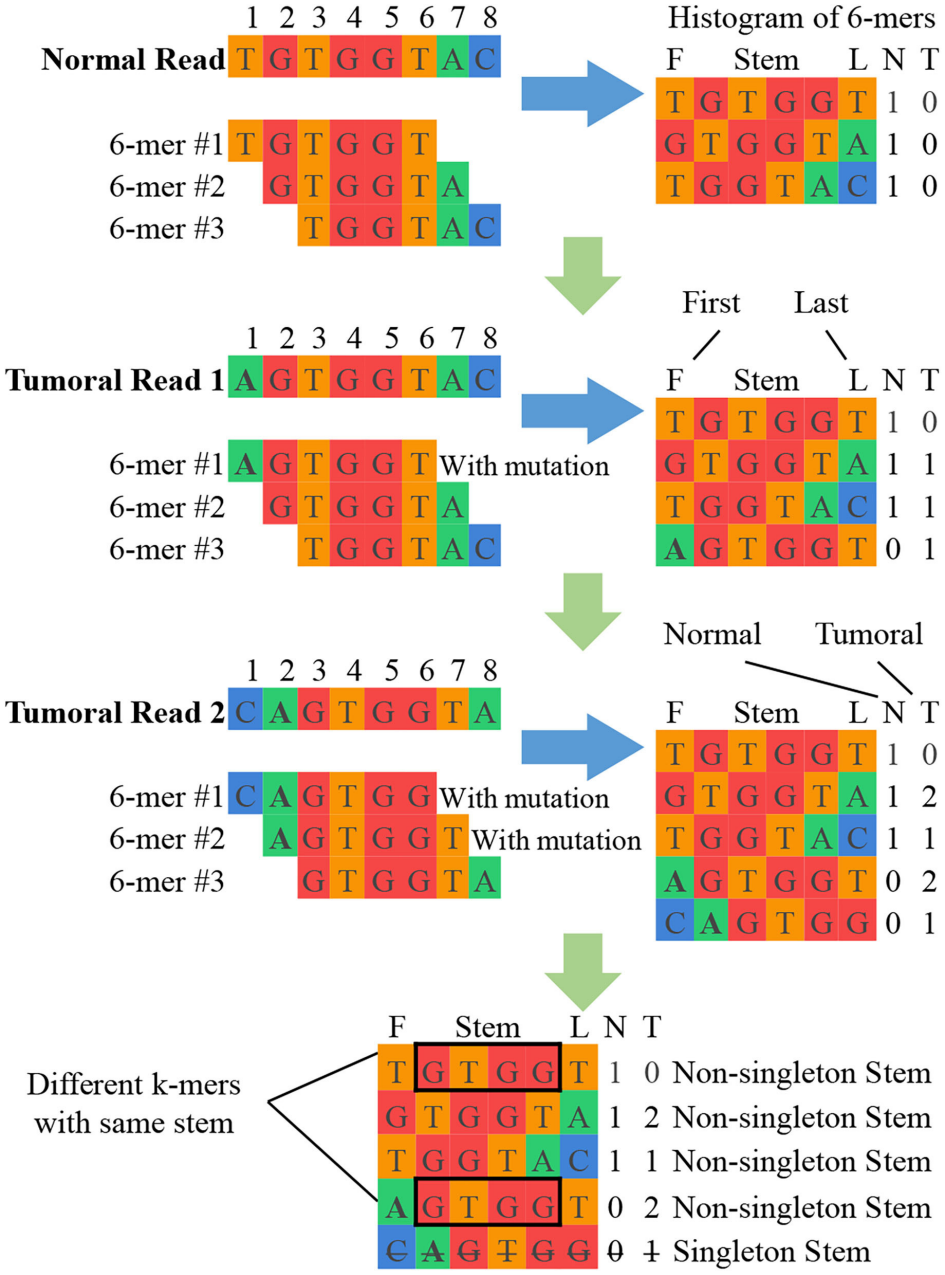
Simplified example of SMUFIN candidate break-point detection. In this example, the stems GTGG and TGGT are potential point of divergence, and their neighboring bases are candidate break-points for mutations.

SMUFIN solves the previously described mutation detection problem for genomics by using k-mer counting to find interesting k-mers and then aligning reads along shared interesting k-mers to reconstruct mutations with their local context. SMUFIN groups k-mers by their middle *k* − 2 characters, or *stem*. Different k-mers with the same stem likely mark the beginning or end of a mutation. Additionally, to improve the effective coverage of the reads, SMUFIN groups together k-mers of reverse complement stems under a canonicalized version of the stem, or *root*. This canonicalization is done by selecting the first of the stem and its reverse complement in alphabetical order. The hierarchy of k-mers, stems, and roots can be seen in Figure 1. Conceptually, SMUFIN is organized into three phases, called *units*:

**K-mer counting:** This unit takes sets of normal and tumoral DNA reads as input and produces a histogram of k-mer counts for each set of reads. At the end of this unit, imbalances in the normal and tumoral frequency indicate a candidate break-point for a mutation. Figure 2 shows a simplified example of k-mer counting. SMUFIN uses *k* values in the range of 24 < *k* < 32. According to the domain experts involved in the original algorithm construction, this range of k-mers is unique enough to accurately align to genomes, and at the same time general enough to accurately pinpoint mutations. For values of k outside this range, results might become either too general (for *k* < =24) or too selective (for *k* > = 32), producing results with poor sensitivity and specificity.

**Labeling:** This unit constructs the *interesting read and k-mer database*. To determine if a read and its k-mers are interesting, each k-mer in the read must be looked up in the k-mer histogram along with each k-mer with the same root. Here, a root-indexed histogram offers data locality for looking up all the k-mers of the same root.

**Figure 3:**
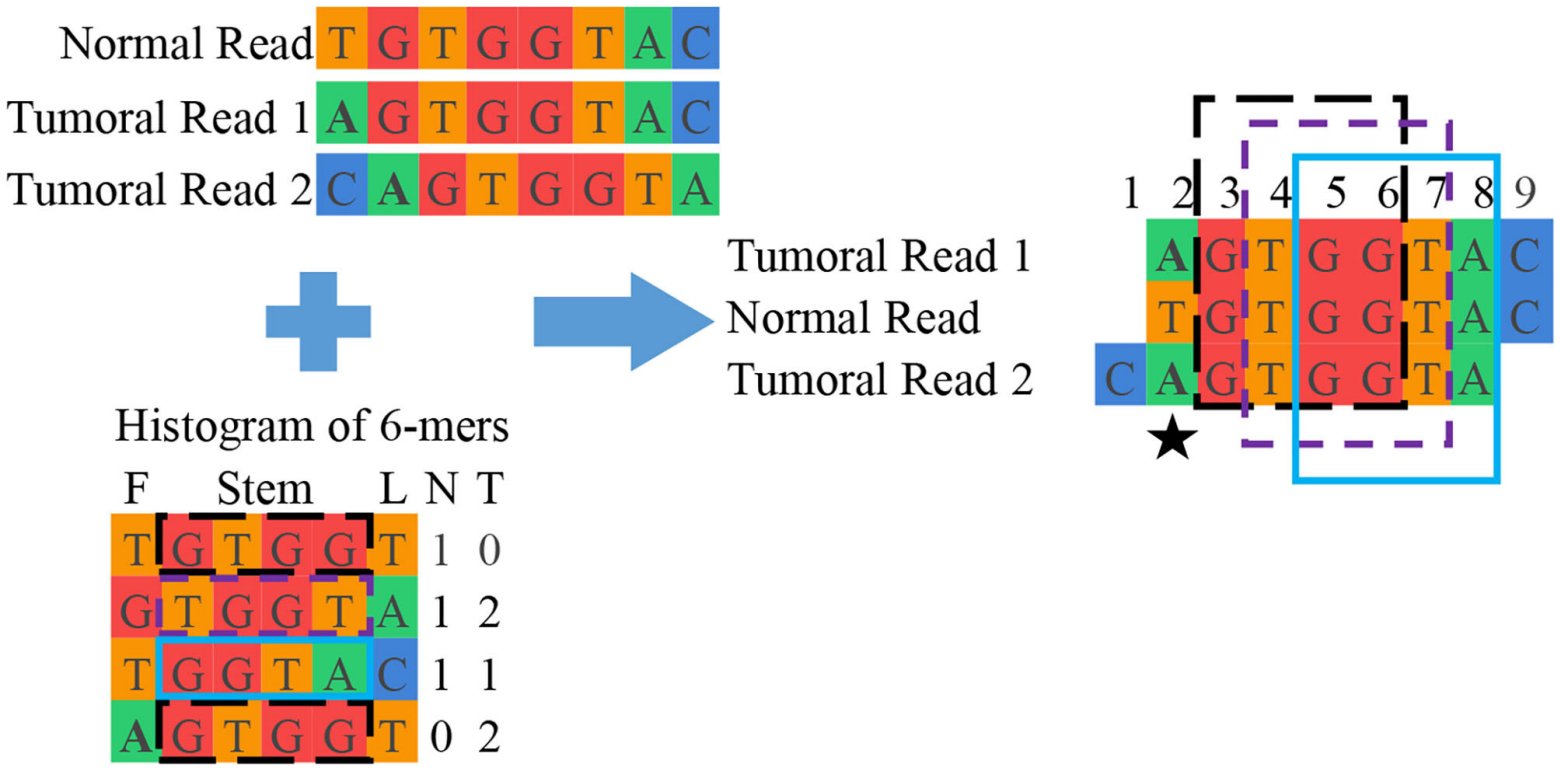
Simplified example of SMUFIN candidate break-point reconstruction. In this example, the second base of the aligned block (⋆) is a candidate break-point for a single point mutation, where a single nucleotide base is changed. In reality, a candidate break-point might be the beginning or end of a larger mutation, such as a structural mutation or a virus.

**Figure 4:**
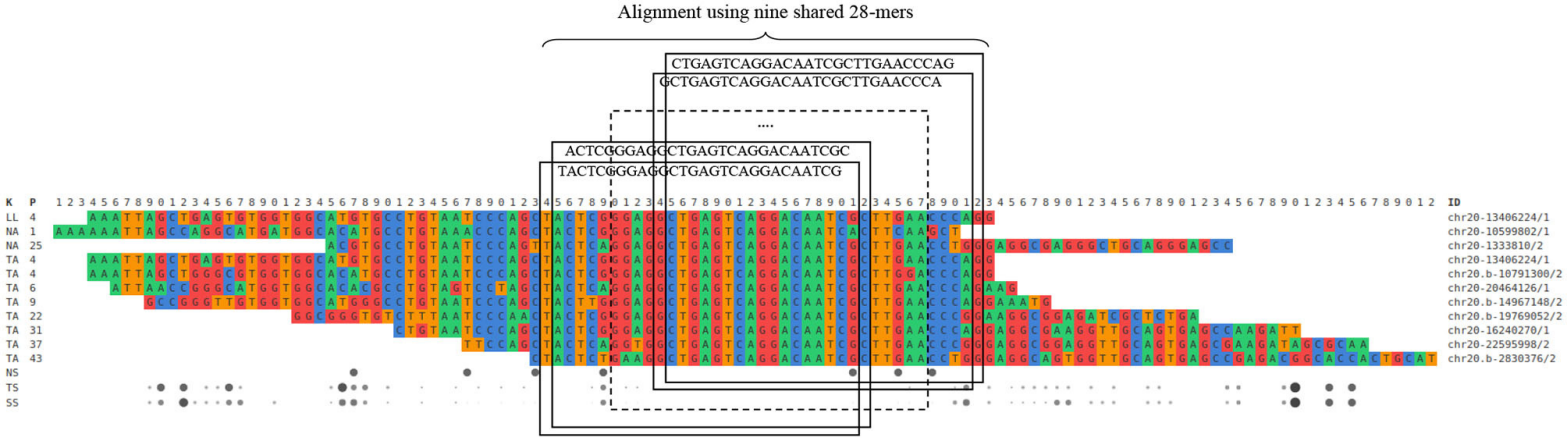
Excerpt of a real SMUFIN output relative to the reconstruction of a candidate break-point using 28-mers.

**Grouping:** This final unit groups reads from normal and tumoral DNA to reconstruct candidate mutations. This is done by looking into the interesting read and k-mer database and clustering reads containing the same interesting k-mers. Once the groups of reads are assembled, they are aligned with each other. Figure 3 shows a simplified example of grouping while Figure 4 offers an excerpt of a real final output of SMUFIN relative to the reconstruction of one candidate break-point.

#### 3.2.1. Implementation Issues

In a typical genome sequenced by next generation sequencing, each part of the DNA appears in tens of different reads; resulting in a sample data set with billions of reads (Illumina, 2019). For instance, a typical uncompressed SMUFIN input is around 740 GB, and it contains approximately 78 billion different roots. With as many different items, the size of a root histogram is almost 10 TB of memory. However, many of these roots appear in only one read, i.e., *singleton* roots, usually considered sequencing noise, e.g., sequencing errors (Bromage and Conway, 2011). The SMUFIN method throws out all the singleton roots reducing the size of the histogram down to around 1 TB, which is still larger than the amount of memory most enterprise machines offer.

**Figure 5:**
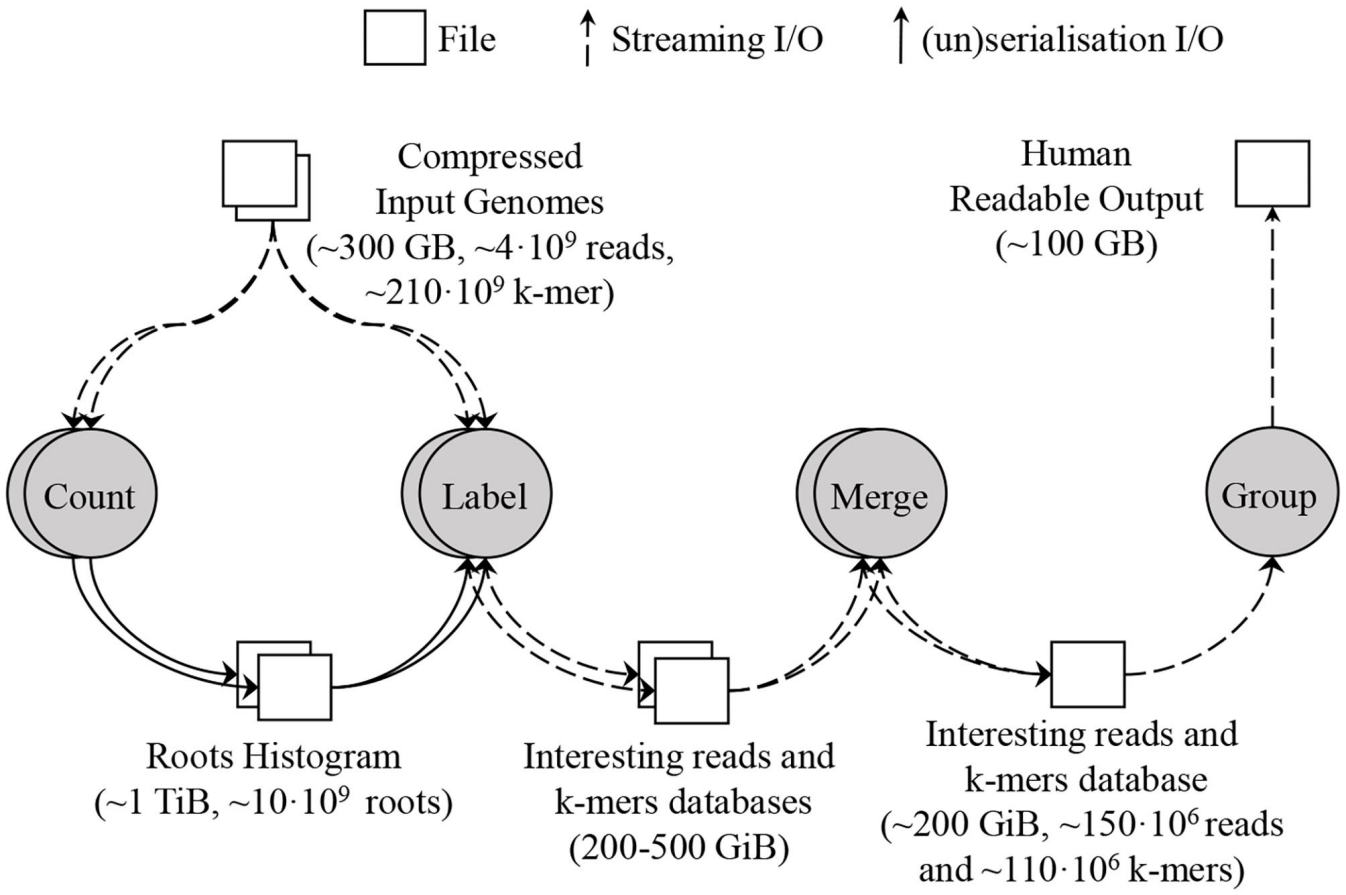
SMUFIN baseline architecture: overview of units and its data flow. In this example, the applications are executed using two partitions; thus Count and Label units are executed twice, reading the whole input genomes twice for each unit.

To deal with this amount of data, SMUFIN supports partitioning the root space into disjoint chunks, and running the Count and Label units of k-mers on a single partition at a time. Using root partitions in the Label unit produces multiple databases, one per root partition. To combine these databases into a single database, a new Merge unit is required which simply concatenates the k-mer tables together and makes a union of the information for the reads with the same ID in each read table. For a typical execution, this database contains up to 150 million reads and 110 million k-mers, and its size is around 200 GB. Figure 5 shows the units and dataflow of SMUFIN assuming the Count and Label units are partitioned. The root partitions can be executed sequentially or in parallel across machines. In the results section, we compare different configurations using the aggregated metrics of all nodes used. Due to the memory requirement of the Count and Label units, they are typically executed using enough partitions to obtain an effective overall DRAM capacity of 2 TB across partitions. For example, when running on servers with 512 GB of DRAM we would split Count and Label into four partitions, each requiring a full pass through the entire input data set. The algorithm takes 12 h to finish using 512 GB of DRAM; 22 h using 256 GB and 8 partitions; 40 h using 128 GB and 16 partitions; and does not finish in a reasonable amount of time with only 32 GB of DRAM and 64 partitions.

The software implementation of the Count unit has been accelerated using GPUs (Cadenelli et al., 2017) and FPGAs (Cadenelli et al., 2019) to improve the time- and energy-to-solution. However, these implementations still require 100s of GB of DRAM for both Count and Filter units. To overcome this, we have also studied the effect of relying on virtual memory management to deal with a small amount of DRAM. We configured the system to use 32 GB DRAM backed by a swap space on two M.2 NVMe drives. On such a system, SMUFIN did not finish in a reasonable amount of time, i.e., several days.

### 3.3. Proposed SMUFIN-F Architecture

**Figure 6:**
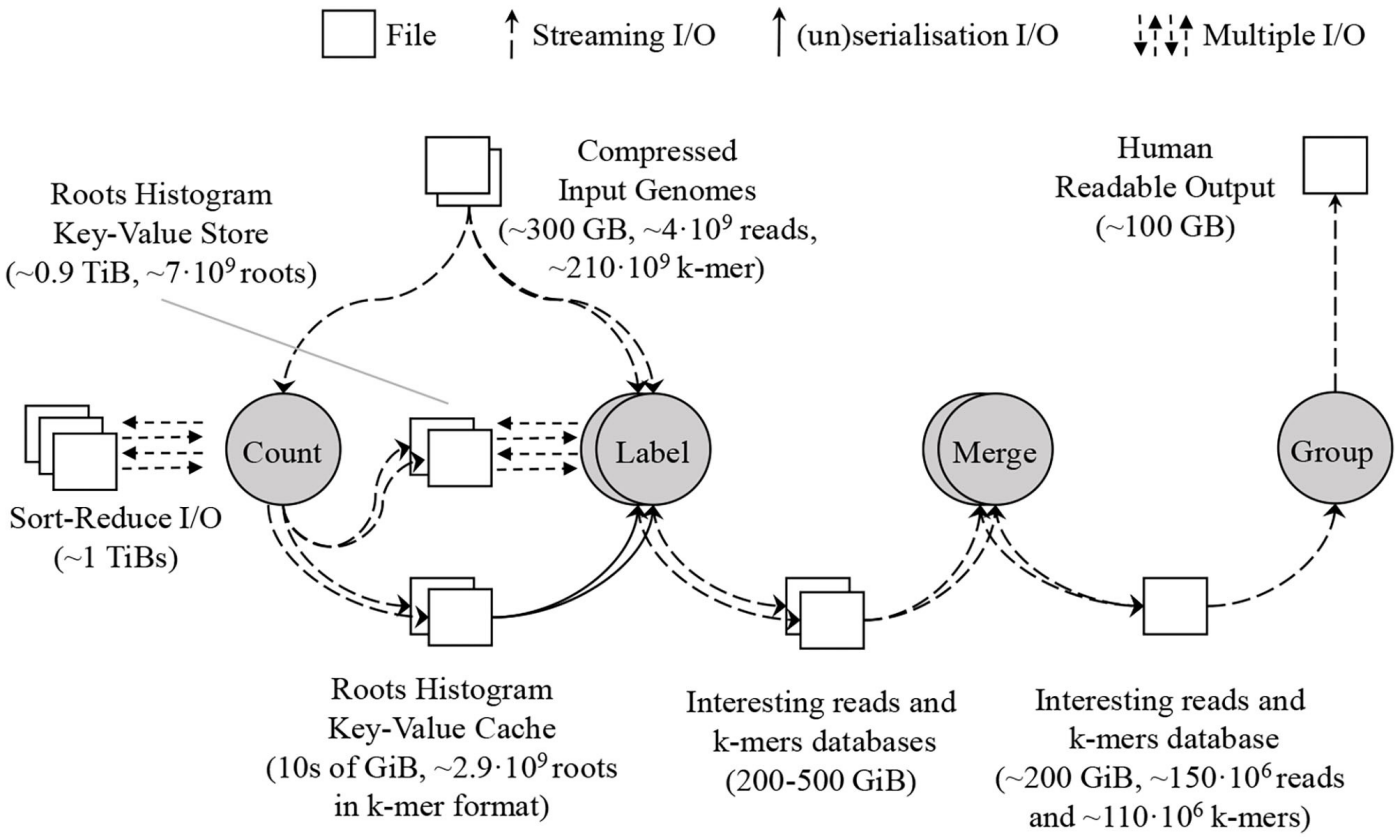
Proposed SMUFIN architecture using SMUFIN-F. The DRAM requirement of the most demanding Count and Label unit is reduced to 10s of GB leveraging Sort-Reduce in the former unit and a key-value store in the latter. The *interesting reads and k-mers database* are untouched.

We have designed SMUFIN-F, a new implementation of the original SMUFIN algorithm, such that it uses only a small amount of DRAM (say 32 GB) and terabytes of NAND-flash storage for intermediate data structures. SMUFIN-F modifies both Count and Label units for optimized storage access. In the Count unit, SMUFIN-F uses the Sort-Reduce (Jun et al., 2017, 2018) algorithm to create a histogram of all k-mers in external storage rather than a hash table in DRAM. In the Label unit, SMUFIN-F implements a key-value store optimized for k-mer access on secondary storage, using a compact, memory resident, and cache-efficient index structure. To reduce the utilization of the key-value store, SMUFIN-F uses an application-specific in-memory cache containing the most-accessed k-mers. After the Label unit, SMUFIN-F uses the same techniques as SMUFIN to build the *interesting read and k-mer database* using RocksDB. The flow of the new implementation is shown in Figure 6.

#### 3.3.1. K-mer Counting With Sort-Reduce

In order to construct a multi-terabyte histogram of k-mers using only a small amount of memory, SMUFIN-F performs k-mer counting in secondary storage using Sort-Reduce (Jun et al., 2017, 2018), which is an algorithm which sequentializes fine-grained random read-modify-writes into secondary storage. Because most flash storage devices have coarse, multi-KB, page-level granularity, updating fine-grained values in secondary storage incurs a large write amplification. Instead, Sort-Reduce collects fine-grained updates in a list and sorts them by location to sequentialize them. During sorting, Sort-Reduce merges update requests to the same location within the request list, without waiting to apply it to the storage. This is similar to compaction of LSM-tree based KV stores (Chang et al., 2006; Google, 2019; RocksDB, 2019), but instead of removing stale items in the case of a duplicate, items with the same key are merged together using a user-defined function. This optimization has significant performance benefits in systems where each location is the target of multiple updates, as in computing histograms or graph analytics.

Since the list of update requests is expected to be much larger than the total capacity of the DRAM, Sort-Reduce uses a two-phase external sorting technique. In the first phase, Sort-Reduce repeatedly brings in blocks of key-value pairs as big as the available DRAM, sorts them, merges requests to the same destination, and then stores the sorted block back in external storage. In the second phase, it merges several of these sorted blocks to produce a bigger sorted-and-reduced block. The merging process is repeated until the key-value list is completely sorted.

In order to efficiently perform k-mer counting of two read sets in storage, SMUFIN-F first modifies the histogram data structure of SMUFIN to make it more storage-efficient. Since the root histogram format used in SMUFIN requires 64 counters (normal and tumoral counters per each of the 16 k-mers in both stems of the root) per entry and it contains 0 s for most of the counts, we can reduce the storage requirement of the histogram by not storing these zero entries. SMUFIN-F accomplishes this by creating a histogram indexed using k-mers, instead of roots. Using this format, every entry of the histogram is made of just two values, normal and tumoral, rather than the 64 using the root format. To maintain the locality of k-mers with the same root, the k-mers are sorted using a comparison function that compares the root first. The implementation of this comparison function is done by producing an integer key for each k-mer where sorting on that key results in the desired ordering for all the k-mers. Using k-mers as the index instead of roots reduces the size of the histogram by as much as 90%.

Sort-Reduce is used to construct a k-mer indexed histogram using k-mers as keys and a pair of counters for each k-mer as values. The two counters represent k-mer counts from the normal sample and the tumoral sample. Each read is split into k-mers, and a histogram update request for each read k-mer is entered into Sort-Reduce. During Sort-Reduce execution, the list of requests is sorted by a k-mer using a custom comparison function grouping k-mers of the same root next to each other. Whenever requests to the same k-mer are discovered, they are merged by adding together the counters of each entry.

It should be noted that while SMUFIN-F uses flash storage instead of memory for processing, flash lifetime is not a serious issue due to the read-intensive nature of the Sort-Reduce algorithm. Sort-Reduce is the only component of SMUFIN-F which writes to storage, and thanks to the effectiveness of merging, we measured the actual amount of intermediate data written to be an order of magnitude less than the original data, matching the observations from GraFBoost (Jun et al., 2018).

#### 3.3.2. Key-Value Store for Histogram Look-Up

To determine if a read and its k-mers are interesting for the SMUFIN method, the Label unit performs a look-up in the histogram for each k-mer in each read. The original SMUFIN implementation uses a hash table to store the histogram in DRAM, but naively moving this main data structure from DRAM to storage incurs a heavy performance penalty. When using a single NVMe drive with 850 K IOPS, performing a single look-up in flash storage for each k-mer is projected to be 11.7x slower than SMUFIN's Label unit. Even when using four NVMe drives in parallel and filtering out singleton k-mers perfectly, we only get an ideal performance of 2x slower. This is an optimistic projection based on the nominal SSD throughput and the assumption that singleton k-mers can be filtered without flash accesses. In reality, we would see lower performance.

To implement the histogram with significantly less DRAM, we constructed a key-value store in flash storage taking advantage of the following application-specific properties of the histogram and how it is used:

1. We take advantage of the fact that the histogram is fully constructed and inserted completely into a key-value store before being accessed. We implement a compact, read-optimized index structure for immutable storage-resident data using a page-granularity multi-level index.
2. We know that approximately 30% of the roots are *singleton* roots, and thus lead to negative look-ups in the Label unit. To expedite such negative searches, we construct a Bloom filter, as well as a band-pass filter during the histogram construction to discard these singletons.
3. We know a histogram's most commonly accessed entries, because the values in the histogram represent the number of look-ups for each entry. Using this information we can statically construct a very efficient cache (in DRAM) to store these entries.

**Figure 7:**
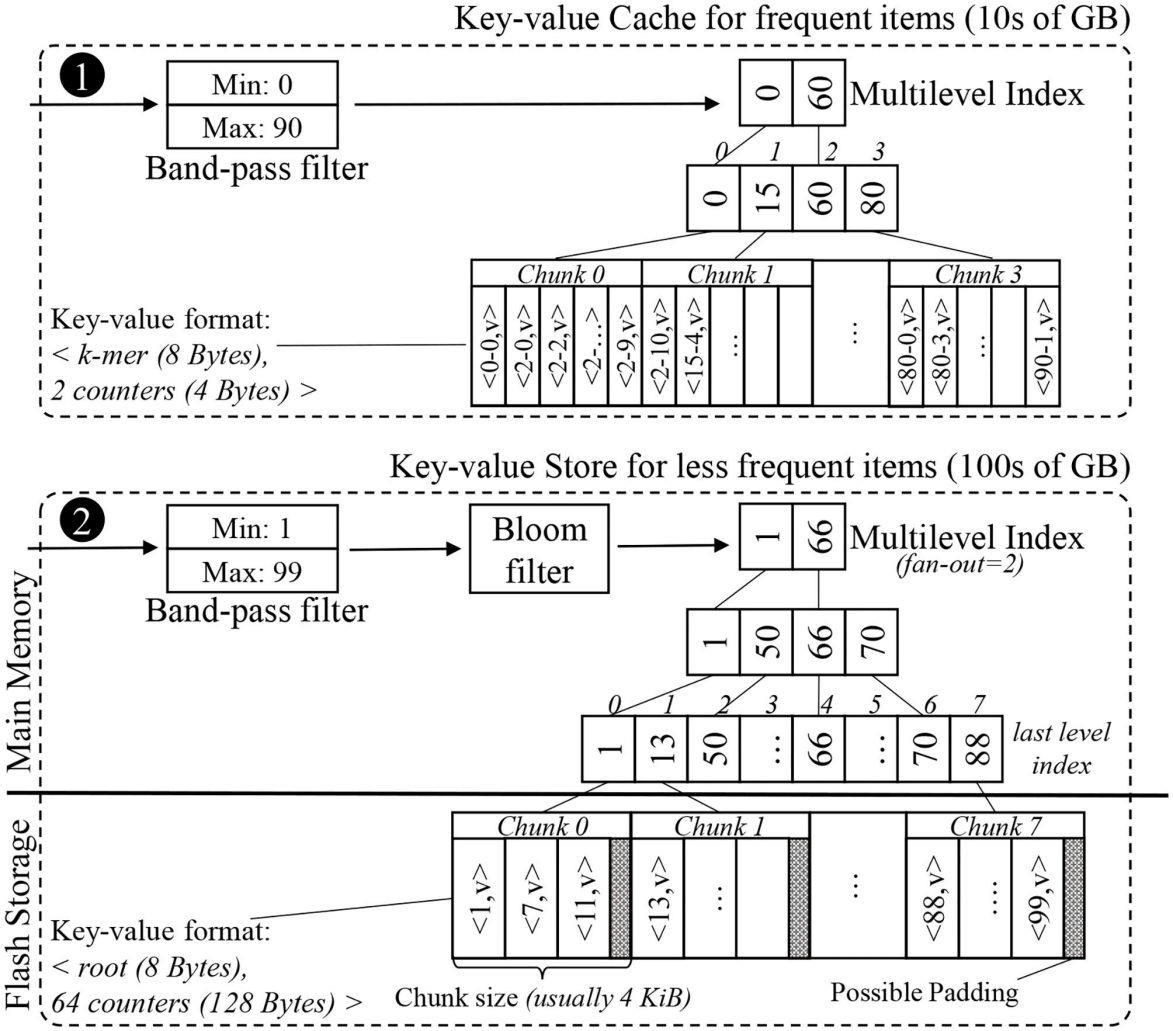
Simplified example of the internal data structures of the proposed key-value cache and store. The key-value format of the items differs between the cache and the store. While the cache uses the space-efficient k-mer format, the store uses the root format to limit each look-up to just one chunk.

Using these insights, we constructed a key-value store and a key-value cache for the Label unit. The overall structure of the in-memory key-value cache and the external key-value store can be seen in Figure 7. This application-optimized key-value store and cache may also be useful in other genomics and n-gram based applications (e.g., matching records from disparate databases, plagiarism checks, and spam filtering).

The histogram is a sorted list of key-value pairs. A data structure that points to each item individually would be too large to fit in DRAM, so we split the list into an array of consecutive fixed-size chunks, and create a much smaller indexing data structure to find the chunk in which a key resides. A good size for chunks is the size of a flash page, i.e., 4 KB, so accessing a chunk only requires one access to flash. We can avoid storing pointers to these chunks by creating an array of keys where the array indices match the chunk indices, and each key is the first key found in the corresponding chunk. For 1 TB of key-value data, this array of non-singleton roots has about 10 million elements, and can be completely memory-resident. We note that this data structure is viable for SMUFIN-F because there are no updates into the histogram after the k-mer counting has completed.

Since the index array is much larger than the L3 cache on a typical processor, each level of the binary search will typically cause a cache miss, bringing in an entire cache line typically for just a single value. One way to reduce cache misses is to create a multilevel-index with a fixed fan-out *k*, e.g., 16. Each level of this k-ary tree uses implicit indices for the next level of the index, i.e., the *ith* key in one level's array corresponds to the range of keys from *k* · *i* to *k* · (*i* + 1) − 1 in the level array below. By using *k* consecutive keys for the search in each level, a single cache miss will bring multiple keys, useful for the search. This will reduce the overall cache misses observed per look-up and will improve the performance. Since this indexing structure uses array indices as implicit pointers to the next level of the index, there is no need for pointers as in a B+ tree. An example multi-level index structure can be seen in the lower half of Figure 7.

To expedite negative look-ups, we first use a *band-pass filter* that removes look-up requests that fall outside the range between the lowest and highest keys seen during the construction of the key-value store. The band-pass filter is simple, but effective, especially when the root set is partitioned into many shards. We then use a *Bloom filter* which is populated during the construction of the key-value store. While Bloom filters are commonly used in many key-value stores, it is especially effective for this case because the table is read-only and there is no need to keep track of deleted items nor to rebuild the filter periodically.

In order to reduce the number of secondary storage access, we also implement an in-memory application-specific key-value cache. There are a few items in the histogram that have very high frequency—typically 5% of the items account for up to 25% of all the positive look-ups. This is due to the fact that there are patterns of repeated nucleic acid sequences (DNA or RNA) in a typical genome (Lander et al., 2001; de Koning et al., 2011). We construct a small, but highly effective in-memory cache using our application-specific knowledge of the histogram's most commonly accessed entries based on its counter values. Entries with the largest counter values will be accessed the most. Just like the storage-resident key-value store, the cache is constructed only once after the histogram construction. The structure of our key-value cache can be seen in the upper half of Figure 7.

Since the data is stored in the exact form necessary for use, this format is actually more efficient for look-ups and usage.

On the other hand, the in-memory key-value cache needs to be as compact as possible because it all goes in DRAM. Therefore, the histogram is stored in the same compact k-mer indexed format used by Sort-Reduce in the Count unit of SMUFIN-F. The keys in the multilevel-index remain as roots to simplify the look-up process because, despite the k-mer-based format used for the histogram, look-ups are still trying to get all the counters for a given root.

## 4. Results

This section compares the performance of the baseline version of SMUFIN on the costly HPC/enterprise-class server machines with 100s of GB of DRAM against SMUFIN-F on a cheaper commodity PC with flash storage described in section 2 and summarized in Table 1. The results show that the proposed method offers significant cost- and power-performance benefits against the state-of-the-art on more expensive hardware. Besides, this section also offers additional experiments to highlight the benefit of asynchronous I/O.

### 4.1. SMUFIN vs SMUFIN-F

**Figure 8:**
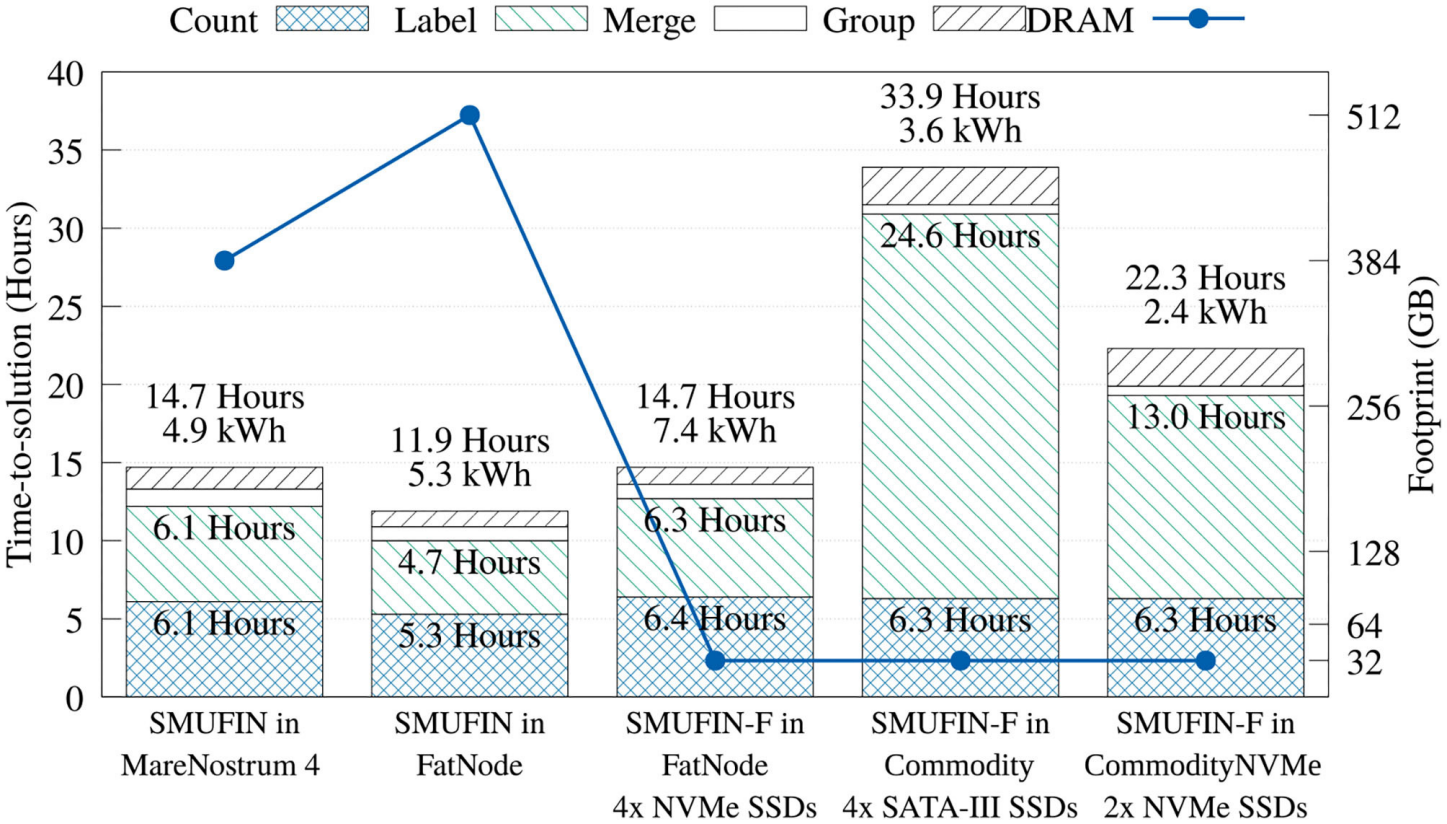
Time-, energy-to-solution, and memory footprint of the entire SMUFIN and SMUFIN-F pipeline on different hardware configurations.

Figure 8 shows that the time in the Count unit is not significantly degraded with the SMUFIN-F implementation that uses Sort-Reduce. Compared to Marenostrum 4, the memory footprint is reduced 12-fold, with a loss of performance of only 4%. Compared to the baseline on FatNode, both proposed SMUFIN-F executions are able to achieve around 70% of its performance while reducing the memory capacity 16-fold.

Regarding the Label unit, the SMUFIN-F implementation using 32 GB of DRAM on FatNode achieves 97 and 75% of the performance of the baseline SMUFIN running on Marenostrum 4 and FatNode using all available DRAM, respectively. This high performance retention is largely due to our application-specific cache, which was able to accommodate 95% of reads in 160 GB, or five partitions of 32 GB. On the other hand, when executed on Commodity, with much slower storage and a fourth of the CPU threads, the performance of the SMUFIN-F Label unit is 5.2x slower than baseline SMUFIN running on FatNode. System traces indicated that the SATA III SSDs are the bottleneck, and faster NVMe drives significantly improved read bandwidth from a steady 800 MiB/s to peaks of 1.5 GiB/s, and reduced time consumption of the Label unit from 24.57 to 13.40 h. This upgrade reduces the overall execution time from 33.9 h to around 23, improving the energy-to-solution from 3.6 to 2.4 kWh.

Regarding the Merge and Group units that were left unchanged, Figure 8 shows that running these units in systems with less DRAM and a slower CPU yields a marginal performance loss, if compared to other units. Here, the only noteworthy difference is that the Group unit, which is I/O intensive, is slower with the reduced bandwidth of the SATA-III SSDs.

In terms of overall performance, FatNode, Commodity, and CommodityNVMe, all using only 32 GB of DRAM, were able to achieve 81, 35, and 53% of performance compared to FatNode with 512 GB of DRAM, respectively.

#### 4.1.1. Energy Consumption and Cost

**Figure 9:**
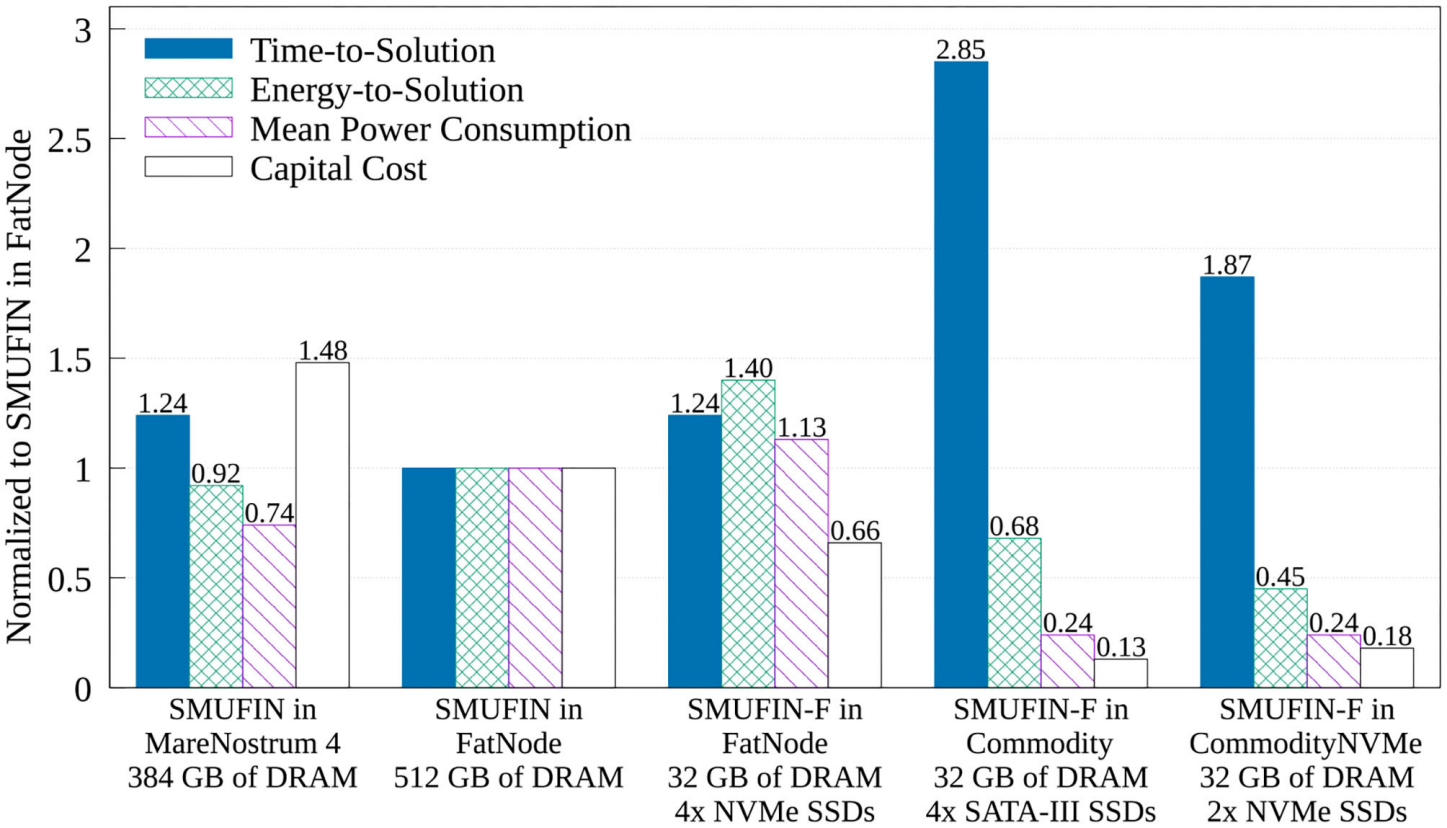
Normalized (to SMUFIN on FatNode) time-to-solution, energy-to-solution, mean power consumption, and capital cost of SMUFIN and SMUFIN-F. Note that since SMUFIN-F in FatNode uses only 32 GB of DRAM instead of the 512 GB needed by SMUFIN, its capital cost is significantly lower.

The true benefit of SMUFIN-F is in its power and cost reduction. In fact, Figure 8 also reports that while SMUFIN-F on CommodityNVMe is 1.87x slower than SMUFIN on FatNode, it also consumes only 45% of the energy to completion. Even the slower Commodity, while being 2.85x slower, consumes only 68% of the energy to completion. During execution, the peak power for FatNode was 549 W while the two commodity PCs only reached 120 W. These results are summarized in Figure 9, which shows the capital cost, power consumption, and energy-to-solution of each system configuration, normalized to SMUFIN on FatNode. It shows that not only are SMUFIN-F-based solutions more affordable, but they also consume significantly less energy per patient.

From a ROI point of view, SMUFIN-F on CommodityNVMe, a PC that costs only 18% of FatNode and requires only 45% of the energy, is only 1.87x slower that the baseline. This cost differential means we could use two CommodityNVMe to process two patients' data in parallel, in less time that it would take on one FatNode. Today, this would require an investment of only 36%, i.e., around 3,300 USD rather than 9,200 USD, and it would consume only 45% of the energy compared to FatNode, 4.8 kWh against 10.6 kWh for every two patients.

### 4.2. Key-Value Store Performance

To evaluate of the performance of our external key-value store we perform two additional experiments:

#### 4.2.1. Comparison Against RocksDB

**Figure 10:**
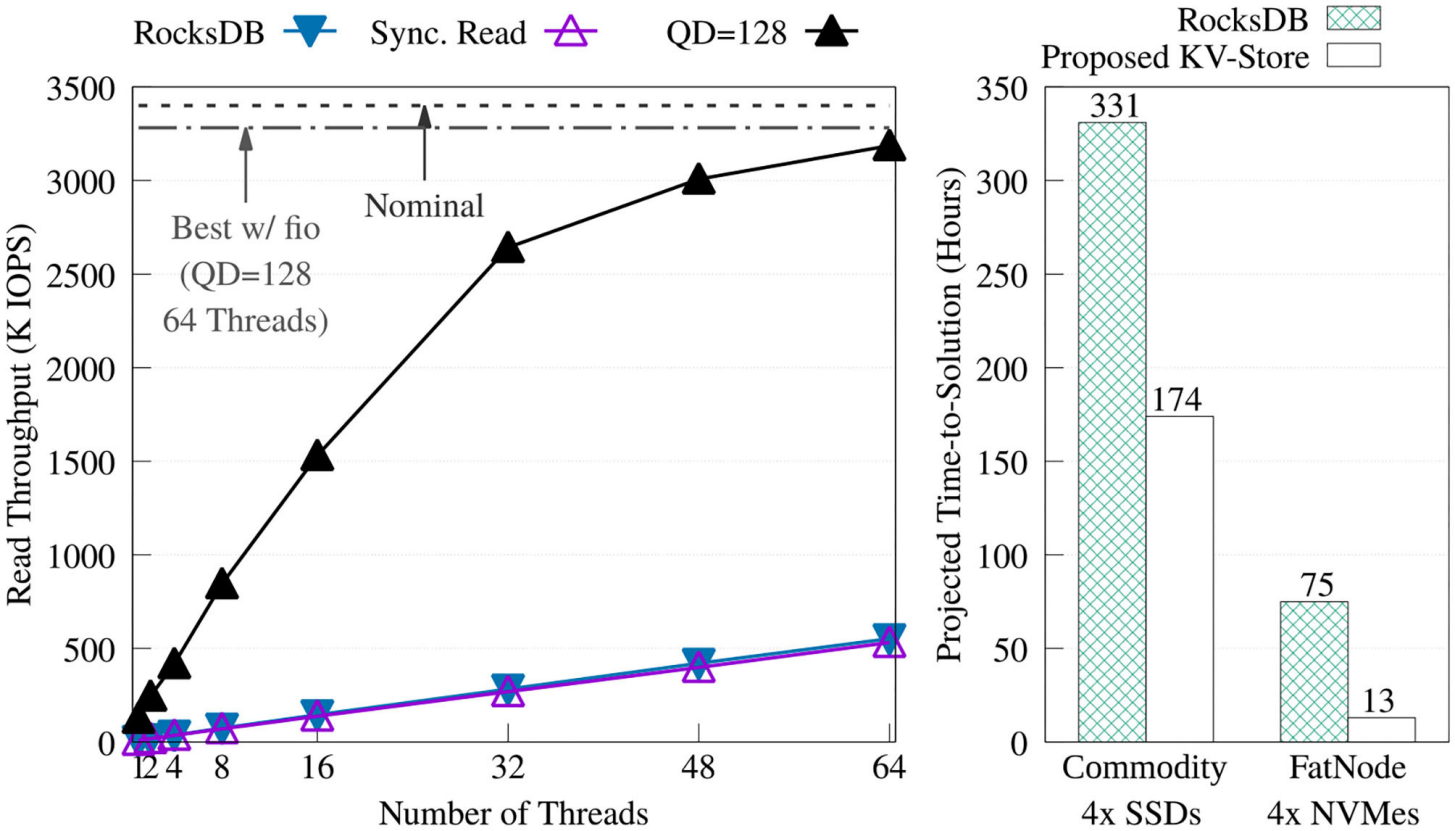
Throughput comparison of the proposed key-value store (without the KV cache) vs. RocksDB 5.15.10 on FatNode with four NVMes **(left)**. Projection of the time-to-solution to process the required 149 billion positive look-ups—one per each non-singleton k-mer—during the Label unit at the maximum throughput achieved by both key-value stores on FatNode and Commodity **(right)**.

We compared our key-value store against RocksDB. To get the best performance from RocksDB, we created the key-value store by adding the keys sequentially without compression. Then, for each experiment, the database was opened read-only, auto compaction was disabled, and level 0 filters and index blocks were pinned in the cache. Figure 10 offers a comparison of the throughput between RocksDB and the proposed key-value store. The figure shows how RocksDB performs very similarly to our synchronous read implementation; most likely because, in both cases, the read time is dominated by I/O latency.

Figure 10 also shows the projected execution time of the Label unit taking into consideration only the time to perform the 149 billion positive look-ups required for each patient, at the throughput obtained with the benchmark shown on the right. On both machines, this chart demonstrates the benefit of asynchronous I/O and highlights the need for a DRAM cache.

#### 4.2.2. Saturating libaio Bandwidth

We demonstrate the efficiency of our external key-value store without in-memory caching using a synthetic 100% random reads workload, which represents a workload similar to the look-ups of the Label unit. The benchmark was executed using different numbers of CPU threads and using synchronous reads and asynchronous reads with different queue depths (QD). In each run, each thread performed 10 million positive look-ups on a pre-built key-value store of 1 billion items (approximately 127 GB). The sizes of keys and values are set to 8 and 128 B respectively to match SMUFIN. We also compare the results against the nominal throughput and the best throughput of libaio measured using fio to emulate the same loads using random 4 KiB reads.

**Figure 11:**
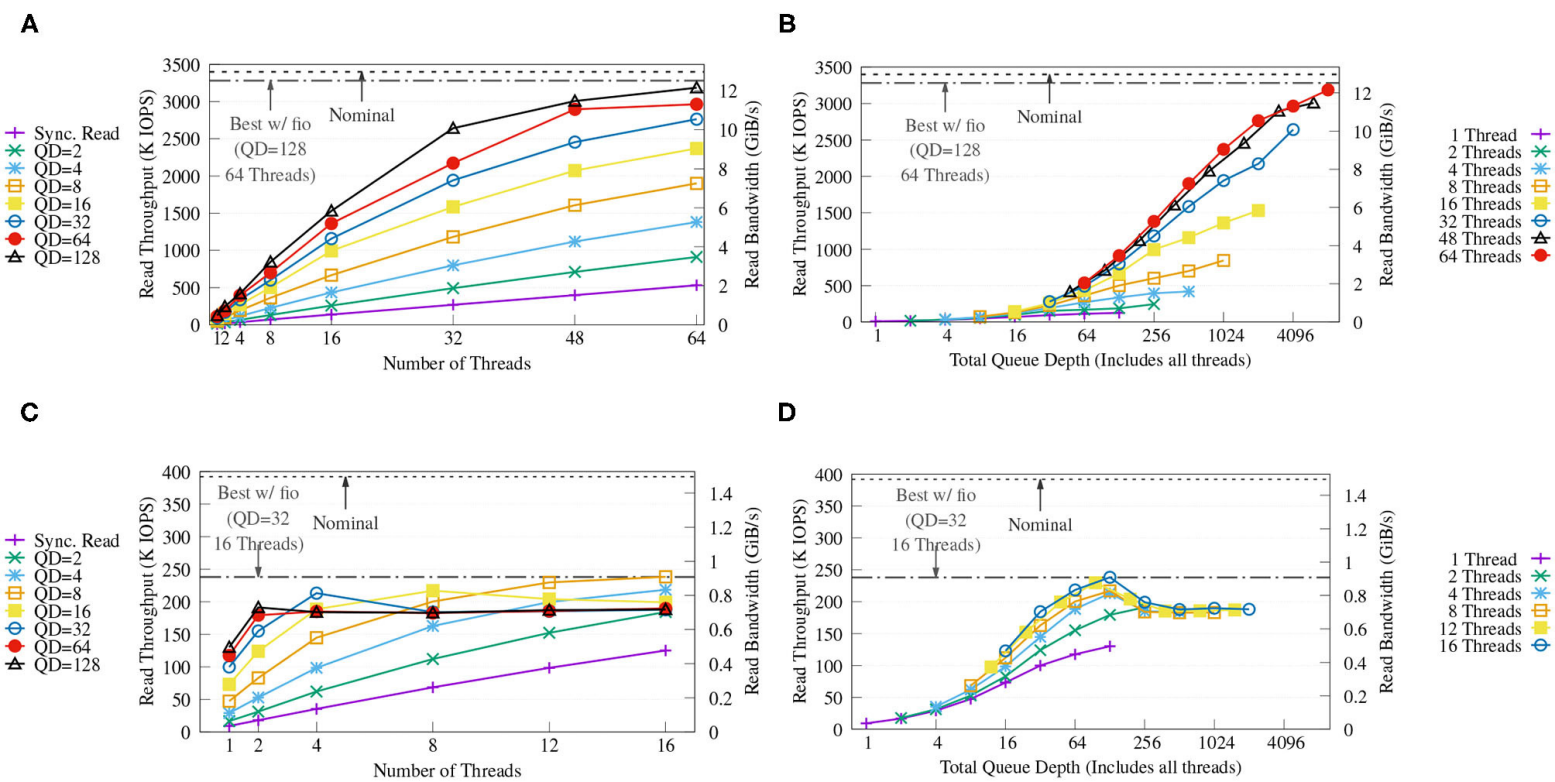
Throughput **(A,C)** and throughput vs. total queue depth of the proposed KV store **(B,D)** on FatNode with four NVMes (top) and on Commodity with four SATA III SSDs (bottom) running a benchmark with variable queue depths (QD) and numbers of threads. For each run, each CPU thread executes 10 million randomized positive look-ups on the same KV store populated with 1 billion items (approx. 127 GB). The horizontal dash and dash-dotted lines report the nominal performance and the best performance achieved with *fio* using *libaio* back-end on the same RAID-0, respectively.

Figure 11A shows that, with four NVMes, our key-value store achieves a maximum throughput of up to 3.1 M IOPS (around 12 GiB/s) with QD = 128 and 64 threads, and that this performance is close to both the performance obtained with fio and the nominal performance of the drives. The figure also displays how both queue depth and number of CPU threads are important to achieve high throughput. Figure 11B shows how the performance increases as the combined queue depth increases, and how a reduced number of threads limits one from benefiting from larger combined queue sizes.

On the other hand, Figure 11C reveals how on Commodity both fio and the proposed key-value store are able to reach only around 60% of the nominal performance of the SATA-III SSDs, possibly due to internal prefetching of the low-cost SSDs and high latency. Increasing QD only provides benefits up to a combined queue depth of 128 as seen in Figure 11D, which is exactly the combined queue depth of the four SSDs used, and deeper queues show diminishing returns.

### 4.3. Key-Value Cache Effectiveness

**Figure 12:**
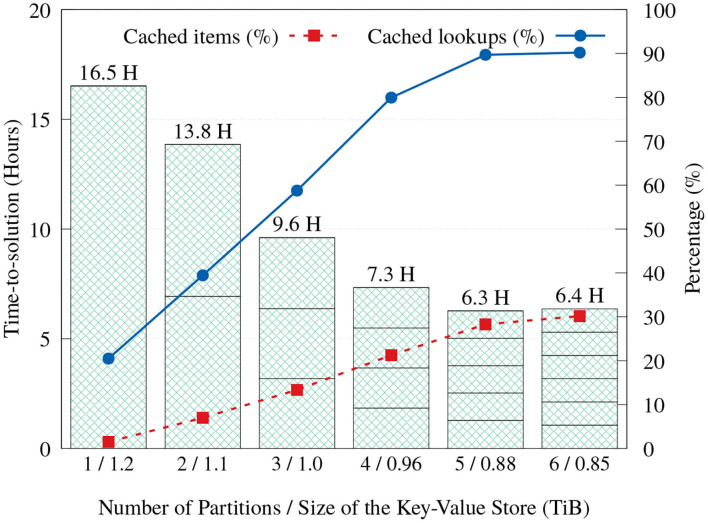
Time-to-solution and cache effectiveness of the proposed Label unit on FatNode with one to six partitions.

As discussed in section 3.3, we implemented a key-value cache that stores items that are predetermined to appear most frequently, in order to reduce the number of storage accesses and reduce execution time. Cache space can be used even more effectively when execution is divided into partitions. When the Label unit divides its input into multiple partitions and executes them in order, each partition execution will have exclusive access to the whole cache capacity, resulting in a larger total number of elements that can be serviced from the cache. Even though more partitions mean more full scans through the input reads, Figure 12 shows that the aggregated time-to-solution on FatNode generally reduces as more partitions are used, by servicing more look-ups from the cache. This trend continues up to five partitions, but with six partitions the execution time stops improving.

## 5. Discussion

We explored the challenge of reducing the DRAM footprint of a genomics application from 100s of GB to 32 GB, using NAND-flash storage as a replacement. The work was motivated by the need of commodity PCs that are able to perform *in-situ* genome analysis in hospitals and clinics; and by the observations that DRAM is facing scaling problems and its price has not been decreasing as significantly as before. Firstly, we modified the k-mer counting algorithm to take advantage of Sort-Reduce to efficiently build a histogram of k-mer frequency. Secondly, we designed a key-value store and cache, tailored for the random read-only workload of the Label unit. We demonstrated via benchmarks how asynchronous I/O and multiple threads are key to extract the performance of flash storage. Results and projections showed how their impact is enormous on workloads with billions of look-ups such as genomics applications.

We were able to reduce the system requirements for the entire SMUFIN-F genomics pipeline to the point that it can run to completion on an affordable commodity PC with 6-core i7 and 32 GB of memory, something the existing SMUFIN implementation cannot. On this PC, SMUFIN-F is 1.87x slower than the enterprise server machine with four times as many cores and 512 GB of DRAM. This commodity PC costs only 18% of the cost of the enterprise server we used and requires only 45% of the energy per patient. As a result, a cluster of SMUFIN-F systems running on multiple commodity PCs costs only 36% as much as a bigger enterprise-class server and consumes only 45% the energy while also slightly improving the throughput. This work will help genomics researchers and ease the adoption of advanced methods and pipelines at the clinical level, which is key to eventually enable precision medicine at large scales.

We believe that a similar approach could apply to other data-intensive applications that scale to multiple nodes just to satisfy the DRAM requirement.

## Data Availability Statement

The raw data supporting the conclusions of this article will be made available by the authors, without undue reservation.

## Author Contributions

A and DC conceived of the presented idea. NC, S-WJ, JP, AW, and A designed the solution, planned the experiments, contributed to the interpretation of the results, and drafted the manuscript. NC, S-WJ, and JP wrote the software and ran the experiments collecting the data. All authors reviewed the results and approved the final version of the manuscript.

## Conflict of Interest

The authors declare that the research was conducted in the absence of any commercial or financial relationships that could be construed as a potential conflict of interest.
